# Donor Heme Oxygenase-1 Promoter Gene Polymorphism Predicts Survival after Unrelated Bone Marrow Transplantation for High-Risk Patients

**DOI:** 10.3390/cancers12020424

**Published:** 2020-02-12

**Authors:** Tomohiro Horio, Eriko Morishita, Shohei Mizuno, Kaori Uchino, Ichiro Hanamura, J. Luis Espinoza, Yasuo Morishima, Yoshihisa Kodera, Makoto Onizuka, Koichi Kashiwase, Takahiro Fukuda, Noriko Doki, Koichi Miyamura, Takehiko Mori, Shinji Nakao, Akiyoshi Takami

**Affiliations:** 1 Division of Hematology, Department of Internal Medicine, Aichi Medical University School of Medicine, Nagakute 480-1195, Japan; kuroro@aichi-med-u.ac.jp (T.H.); shohei@aichi-med-u.ac.jp (S.M.); ksakai@aichi-med-u.ac.jp (K.U.); hanamura@aichi-med-u.ac.jp (I.H.); 2 Hematopoietic Cell Transplantation Center, Aichi Medical University Hospital, Nagakute 480-1195, Japan; ykodera@river.ocn.ne.jp; 3 Department of Clinical Laboratory Science, Kanazawa University School of Medical Sciences, Kanazawa 920-0942, Japan; eriko86@staff.kanazawa-u.ac.jp (E.M.); luis1.esp2.cd@outlook.com (J.L.E.); 4 Department of Promotion for Blood and Marrow Transplantation, Aichi Medical University School of Medicine, Nagakute 480-1195, Japan; ymorisim@aichi-cc.jp; 5 Central Japan Cord Blood Bank, Seto 489-8555, Japan; 6 Department of Hematology and Oncology, Nakagami Hospital, Okinawa 904-2195, Japan; 7 Department of Hematology and Oncology, Tokai University School of Medicine, Isehara 259-1193, Japan; moni5@mac.com; 8 Japanese Red Cross Kanto-Koshinetsu Block Blood Center, Tokyo 135-8521, Japan; k-kashiwase@ktks.bbc.jrc.or.jp; 9 Hematopoietic Stem Cell Transplantation Unit, National Cancer Center Hospital, Tokyo 104-0045, Japan; tafukuda@ncc.go.jp; 10 Hematology Division, Tokyo Metropolitan Cancer and Infectious Diseases Center Komagome Hospital, Tokyo 113-8677, Japan; n-doki@cick.jp; 11 Department of Hematology, Japanese Red Cross Nagoya First Hospital, Nagoya 453-8511, Japan; k-miyamura@nagoya-1st.jrc.or.jp; 12 Division of Hematology, Department of Medicine, Keio University School of Medicine, Tokyo 160-8582, Japan; tmori@a3.keio.jp; 13 Department of Hematology, Faculty of Medicine, Institute of Medical, Pharmaceutical and Health Sciences, Kanazawa University, Kanazawa 920-8641, Japan; snakao8205@staff.kanazawa-u.ac.jp

**Keywords:** *HO-1*, unrelated donor, bone marrow transplantation, single nucleotide polymorphism

## Abstract

Heme oxygenase-1 (HO-1), an intracellular enzyme that catalyzes the degradation of heme into biliverdin, free iron, and carbon monoxide, exerts anti-inflammatory and cytoprotective effects against endothelial cell injury. The *HO-1* promoter gene has one important single-nucleotide polymorphism (SNP) rs2071746 (-413A>T) that is functional, and the A allele has been reported to be associated with higher *HO-1* expression levels than the T allele. We investigated the influence of the *HO-1* rs2071746 SNP on the transplant outcomes in 593 patients with hematological malignancies undergoing unrelated, human leukocyte antigen (HLA)-matched, T-cell-replete bone marrow transplantation (BMT) through the Japan Donor Marrow Program. In patients with high-risk diseases, the donor A/A or A/T genotype was associated with better 5 year overall survival (35% vs. 25%; *p* = 0.03) and 5 year disease-free survival (35% vs. 22%; *p* = 0.0072), compared to the donor T/T genotype. These effects were not observed in patients with low-risk diseases. The current findings therefore indicate that *HO-1* rs2071746 genotyping could be useful for selecting donors and tailoring transplant strategies for patients with high-risk hematologic malignancies.

## 1. Introduction

Allogeneic stem cell transplantation (allo-HSCT) is an important therapeutic option to cure advanced non-malignant and malignant hematologic diseases. However, its effectiveness has been limited by mortality and morbidity, due to transplant-related complications, such as conditioning regimen-related organ and tissue damage, severe infection, and graft-versus-host disease (GVHD). Several clinical and experimental studies [1,2,3,4,5,6] have shown that these complications, along with calcineurin inhibitors, activate and damage endothelial cells, potentially leading to organ dysfunction and subsequently multiorgan dysfunction syndrome. Thus, the modulation of endothelial cell injury could help to prevent or treat serious organ dysfunction after allo-HSCT.

Heme oxygenase-1 (HO-1), also known as a 32 kDa heat-shock protein, is an intracellular enzyme that catalyzes the degradation of heme into biliverdin, ferrous iron, and carbon monoxide (CO), with biliverdin being subsequently catabolized into bilirubin [7,8,9,10,11]. *HO-1* is highly induced in response to various stress signals, such as free heme and hemoglobin, inflammatory cytokines, ischemia, endotoxins, irradiation, and mucosal damage [9,10,11,12,13,14,15,16]. *HO-1* thus exerts cytoprotective effects on endothelial cells through ant-oxidative, anti-inflammatory, anti-apoptotic, and anti-thrombotic effects, which are coordinated with heme metabolites of CO and bilirubin.

The *HO-1* gene, also called the *HMOX1* gene, is mainly expressed in monocytes/macrophages, natural killer cells, endothelial cells, and the heart [17]. One important single nucleotide polymorphism (SNP) in the promoter region of the *HO-1* gene, rs2071746 (-413A>T), is functional, and the major A allele is reported to be associated with higher expression of *HO-1* than the minor T allele [18,19]. There is growing evidence to support that non-human leukocyte antigen (HLA) genetic polymorphism represents a significant determinant of outcomes after allo-HSCT [20,21,22,23,24,25,26]. These findings prompted us to investigate the impact of the rs2071746 SNP in the *HO-1* gene on the clinical outcomes of patients undergoing allogeneic bone marrow transplantation (BMT), using an HLA allele-matched, unrelated donor through the Japan Marrow Donor Program (JMDP).

## 2. Results

### 2.1. The Frequencies of HO-1 Genotypes

The *HO-1* rs2071746 SNP was analyzed in 593 HLA-A, -B, -C, -DRB1, -DQB1, and -DPB1 allele-matched, unrelated BMT donor-transplant recipient pairs (Table 1). Based on the disease status and other risk factors that influence post-transplant outcomes, as previously reported [22,27,28,29], standard-risk disease was defined as acute myeloid leukemia (AML), acute lymphoblastic leukemia (ALL), or multiple myeloma (MM) in the first complete remission; malignant lymphoma (ML) in any complete remission; or myelodysplastic syndrome (MDS) or chronic myeloid leukemia (CML) in the chronic phase. Both ALL and AML, which were in the second complete remission, were included in the high-risk group, according to recent reports [27,29] indicating that patients with AML and ALL in the second complete remission have worse post-transplant outcomes than those in the first complete remission. All other conditions were classified as high-risk disease. The genotype frequencies of A/A, A/T, and T/T were 21%, 52%, and 27%, respectively, in recipients, and 23%, 52%, and 25% in donors. These results were in accordance with the Hardy-Weinberg equilibrium (*p* = 0.49), and were similar to the HapMap data in the Japanese population (27%, 50%, and 23%, respectively) [30].

### 2.2. Transplant Outcomes According to the HO-1 Genotype

**Figure 1:**
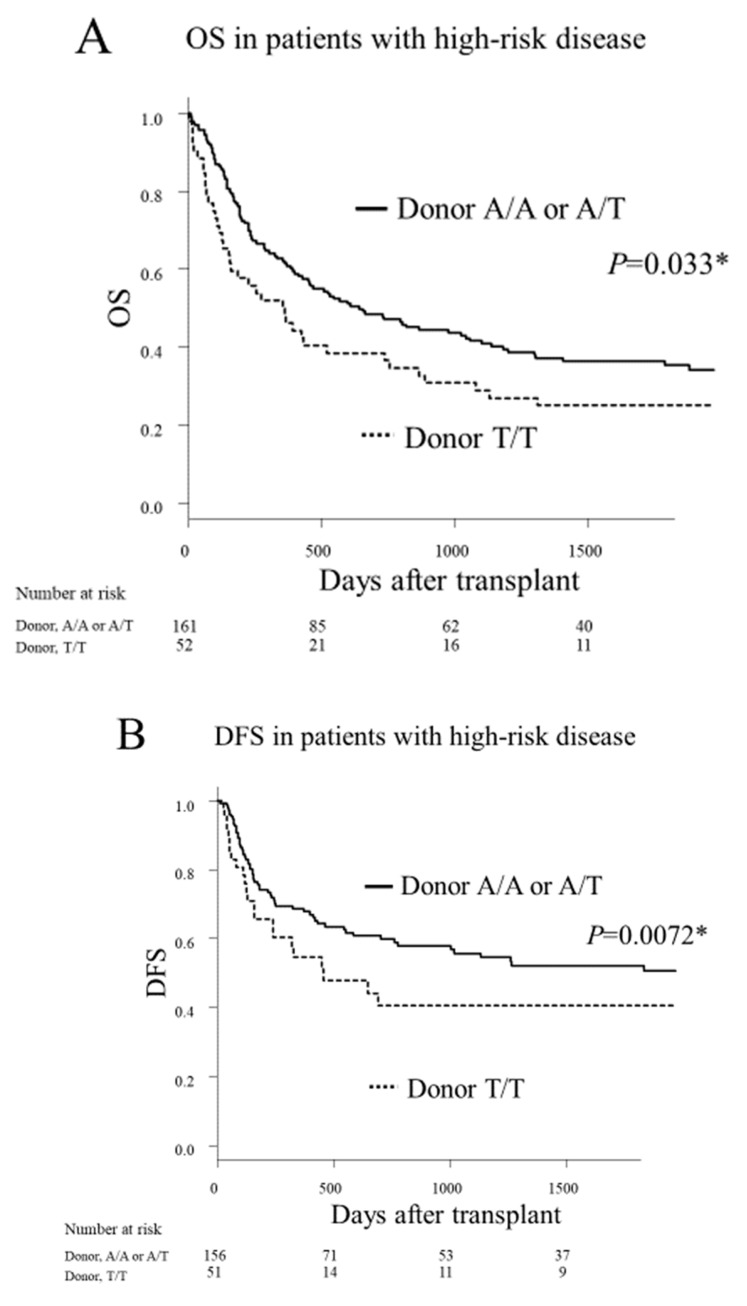
The Kaplan–Meier analysis of the overall survival (OS) rates (**A**) and the disease-free survival (DFS) rates (**B**) after transplantation, according to the donor *HO-1* genotype in patients with high-risk disease. Solid lines represent the donor A/A or A/T genotype. Dashed lines represent the donor T/T genotype. An asterisk (*) denotes statistical significance (*p* < 0.05).

The analysis on the influence of the *HO-1* on clinical outcomes after transplantation was stratified into standard-risk and high-risk disease groups to account for its prognostic significance. In the high-risk disease group (*n* = 232), the donor A/A or A/T genotype was associated with significantly better 5 year overall survival (OS; 35% vs. 25%, *p* = 0.033; Figure 1A) and significantly better 5 year disease-free survival (DFS; 35% vs. 22%, *p* = 0.0072; Figure 1B), compared to the donor T/T genotype. There were no significant differences between the donor A/A or A/T genotype and the donor T/T genotype with regard to the rates of relapse, transplant-related mortality (TRM), and graft-versus-host disease (GVHD; see Tables S1 and S2). In the standard-risk disease group (*n* = 361), the donor genotype had no significant effect on the transplant outcomes (see Tables S1 and S2, Figure S1). The recipient genotype had no significant effects on the transplant outcomes regardless of the disease risk (see Tables S1 and S2).

### 2.3. Multivariate Analysis

Multivariate analyses were performed by adjusting for the cofounding variables, such as recipient, donor, and transplant characteristics, the details of which are described in Section 4.3. In the high-risk disease group, the donor A/A or A/T genotype showed significantly better 5 year DFS (hazard ratio (HR) = 0.64; 95% confidence interval (CI) = 0.44–0.93; *p* = 0.019; Table 2) and tended to show better 5 year OS (HR = 0.72; 95% CI = 0.50–1.04; *p* = 0.082) compared to the donor T/T genotype. In the standard-risk disease group, the donor genotype had no significant effect on the DFS or OS. The donor *HO-1* genotype showed no significant effects on the TRM or GVHD rates in the multivariate analysis (Table 2 and Table 3). The recipient *HO-1* genotype did not significantly influence the transplant outcomes.

### 2.4. Main Causes of Death

**Figure 2:**
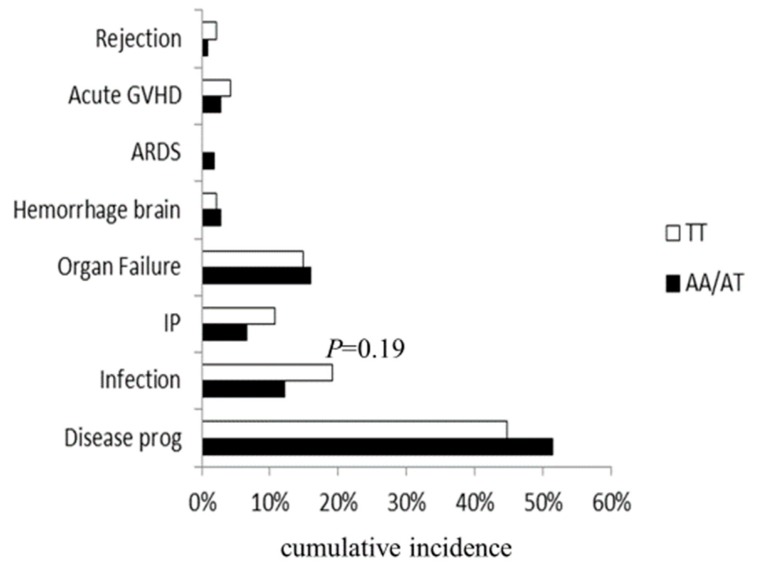
Main causes of death after transplantation, according to the donor *HO-1* genotype in patients with high-risk disease. ARDS: acute respiratory distress syndrome; IP: interstitial pneumonia; disease prog: disease progression.

When the main causes of death were analyzed according to the *HO-1* genotype, although the donor A/A or A/T genotype showed relatively low cumulative incidence of fatal infection in the high-risk disease group (12% vs. 19%, *p* = 0.19; Figure 2), there were no statistically significant differences.

## 3. Discussion

The current study showed that the donor A/A or A/T genotype of the *HO-1* rs2071746 promoter SNP was associated with better DFS than the donor T/T genotype in patients with high-risk hematologic malignancies who received unrelated, HLA-matched BMT through the JMDP. Of note, individuals with the *HO-1* rs2071746 A allele have been suggested to have higher promoter activity, which induces higher expression levels of *HO-1* compared to those without the A allele [18,19], thus suggesting that donor-derived HO-1 may have beneficial effects on survival after allo-BMT. This is the first report to demonstrate that the *HO-1* SNP may be involved in the survival outcomes after allo-HSCT.

The mechanisms through which the donor *HO-1* A/A or A/T genotype (plausibly associated with the higher inducibility of HO-1) exerts its beneficial effects remains to be determined. HO-1 is only marginally produced in the resting state, but is rapidly and highly induced in response to various oxidative stresses (e.g., organ and tissue damage and infection), and acts as an anti-inflammatory and cytoprotective protein [7,9,10,11,15,31,32]. Evidence of HO-1 induction improving the survival outcomes after allo-HSCT has been demonstrated in previous studies using mouse models [31,33]. It was shown that the induction of HO-1 reduced serum levels of proinflammatory cytokines, prevented damage to the intestinal mucosa and subsequent bacterial invasion, and improved overall survival after allo-HSCT [33]. Another study [31], in which transplantation was performed from HO-1 wild-type mice to HO-1 knockout mice, revealed that donor macrophages play pivotal roles in the recovery of the HO-1 function after allo-HSCT. In humans, HO-1 deficiency is associated with a distinctive feature: oxidative stress induces severe endothelial cell damage [10]. Vascular endothelial cell injury after allo-HSCT can potentially cause thrombotic microangiopathy, sinusoidal obstruction syndrome, capillary leak syndrome, and acute lung injury, leading to end-organ dysfunction [1,2,3,4,5,6]. These findings suggest that while organ and tissue damage from the conditioning regimen, infections, GVHD, and calcineurin inhibitors cause oxidative stress, which damages endothelial cells, the same can activate donor macrophages to produce HO-1, protecting against endothelial cell injury and subsequently preventing organ dysfunction. BMT from donors with the *HO-1* A/A or A/T genotype may be associated with improved survival outcomes in recipients by enhancing the beneficial effects of HO-1. However, this hypothesis is highly speculative, because the current study could not provide information on the inducibility of HO-1 or the degree of organ and endothelial cell damage, according to the HO-1 genotype.

Further evidence that the *HO-1* rs2071746 A/A or A/T genotype has the advantage of maintaining the organ function by protecting endothelial cells against oxidative stress may be seen in previous reports [18,19], which have shown that the *HO-1* rs2071746 A/A genotype was associated with a reduced incidence of ischemic heart disease compared to the A/T or T/T genotypes in the Asian adult population, and that the donor *HO-1* rs2071746 A/A or A/T genotype was associated with better graft survival and a better liver function after liver transplantation compared to the donor T/T genotype in the European adult population. On the other hand, one report noted that the *HO-1* rs2071746 A/A and A/T genotypes were associated with an increased incidence of acute kidney injury in the American infant population [34]. A plausible reason for this discrepancy may be the negative effect of hyperbilirubinemia, which possibly results from the higher inducibility of the HO-1 genotype (A/A or A/T) in neonates. Further studies using additional cohorts are needed to better understand this discrepancy.

Recent reports [35,36,37] have suggested that the overexpression of *HO-1* may promote the progression and relapse of various blood cancers, especially CML, as well as carcinogenesis, progression, and resistance to therapy in solid cancers. Unlike these observations, in the present study, the donor *HO-1* A/A or A/T genotype (plausibly associated with the higher inducibility of *HO-1*) was associated with better survival outcomes without increasing the risk of disease relapse after allo-HSCT in patients with high-risk patients. However, allo-HSCT can exert a robust graft-versus-leukemia (GVL) effect [38], which may eliminate the adverse effect associated with the higher *HO-1* inducibility to potentially to promote progression and relapse. This hypothesis may be supported by the current findings that post-transplant progression and relapse rates in patients with CML were comparable between the donor *HO-1* A/A or A/T genotype vs. the donor *HO-1* T/T genotype, with 33% vs. 33% (*p* = 0.82) in the high-risk group, and 21% vs. 42% (*p* = 0.14) in the standard-risk group. Studying the blood samples of donors and recipients would be useful for testing this hypothesis and defining the functional roles of the *HO-1* SNP in post-transplant survival and relapse. Unfortunately, no blood samples of donors or recipients were available in the present study, and the functional roles of *HO-1* on post-transplant outcomes cannot be clarified.

It has been proposed that a higher *HO-1* expression level prevents the progression of GVHD and acts prophylactically on sinusoid obstruction syndrome (SOS), while promoting relapse [39,40,41]. Although the results of this study suggest that the higher expression of *HO-1* is associated with favorable survival outcomes after allo-HSCT, the higher expression of *HO-1* has not been shown to affect the risk of disease relapse or the development of GVHD. Furthermore, this cohort did not include information on the incidence of SOS, so the association between *HO-1* and SOS is unknown. A plausible explanation for these differences may be due to the fact that the higher expression of *HO-1*—which was derived from the donor, not from the patient—appeared to influence the better survival outcomes after allo-HSCT. Namely, *HO-1* derived from the vascular endothelium may be involved in the suppression of GVL effects and GVHD development, whereas *HO-1* derived from donor blood cells may be mainly responsible for its anti-inflammatory and cytoprotective effects, leading to the prevention of organ damage. However, contrary to this hypothesis, a recent report from Denmark [39] showed that higher *HO-1* expression derived from the donor may be associated with a higher risk of relapse after allo-HSCT, based on an investigation of the (GT)_n_ repeat in the promotor region of donors and recipients receiving allo-HSCT. Differences between the Japanese and Danish ethnic groups might account for the role of *HO-1* in the induction of the GVL effect. These hypotheses are highly speculative and need to be clarified by future research.

In this study, the beneficial effects of the donor *HO-1* A/A or A/T genotype were only observed in patients with high-risk diseases, and not in those with standard-risk diseases. Although it is unclear what causes this difference, patients with high-risk diseases are often heavily treated in an attempt to achieve remission, and may potentially be prone to endothelial cell damage, which may increase the importance of HO-1 in post-transplant survival. This hypothesis may be supported by previous reports showing that an advanced disease status was associated with an increased risk of endothelial cell damage, such as sinusoidal obstruction syndrome and thrombotic microangiopathy, after allo-HSCT [42,43].

One major limitation of this study is the lack of results that conclude the functional roles of the *HO-1* SNP in survival outcomes after BMT. The association of the surrogate markers for endothelial cell damage after allo-HSCT, such as serum levels of angiopoietin 2 and thrombomodulin [44], with the *HO-1* SNP may offer useful information on this issue. Unfortunately, no serum samples were available in the present study. A second major limitation of this study is that information on transplant-associated thrombotic microangiopathy (TAM) resulting from endothelial cell damage was also unavailable. A study to examine whether the *HO-1* genotype and its expression levels contribute to the development of post-transplant complications (e.g., TAM, organ damage, and GVHD) and the graft-versus-tumor effect is warranted.

## 4. Materials and Methods

### 4.1. Patients

The *HO-1* rs2071746 genotypes were determined on 593 recipients with hematological cancers who underwent transplant via the JMDP with T-cell-replete marrow from HLA-A, -B, -C, -DRB1, -DQB1, and -DPB1 allele-matched donors between January 1993 and December 2007, as well as their unrelated bone marrow donors. The HLA genotyping of recipients and donors was performed by the Luminex microbead method, as described previously (Luminex 100 System; Luminex, Austin, TX, United States) [45,46]. Although the Luminex microbead method does not bring unambiguous HLA four-digit typing for all genotypes, the JMDP has validated that this method can identify all HLA alleles with >0.1% frequency among the Japanese population [47]. Patients with a prior history of any transplantation have been excluded. The final clinical survey of these patients was finalized by November 1, 2008. The diagnoses were ALL in 145 patients (24%), AML in 197 (33%), MDS in 82 (14%), ML in 64 (11%), CML in 101 (17%), and MM in 4 (1%). Cyclosporine- or tacrolimus-based regimens were administered to all patients for GVHD prophylaxis, and anti-T cell therapy (e.g., anti-thymocyte globulin and ex vivo T cell depletion) was administered to none of the patients. All patients and donors gave their written informed consent at the time of transplantation to participate in molecular studies of this nature, in accordance with the Declaration of Helsinki. This project was approved by the Institutional Review Boards of Aichi Medical University School of Medicine (2017-M002) and Kanazawa University, as well as the JMDP.

### 4.2. HO-1 Genotyping

The genotypes of *HO-1* rs2071746 were determined using the TaqMan allelic discrimination method, as previously reported [20]. The genotyping assay was done in 96 well PCR plates, using specific TaqMan probes for the *HO-1* gene single nucleotide variation rs2071746 (C__15869717_10), and in a StepOnePlus Real-Time PCR system (Applied Biosystems, Foster City, CA, United States).

### 4.3. Data Management and Statistical Analysis

The JMDP collected data using a standardized report form [48,49]. Follow-up reports were submitted at 100 days, 1 year, and then annually after transplantation. The pre-transplant cytomegalovirus (CMV) serostatus was routinely checked in the patients only, and not in the donors. Engraftment was defined by an absolute neutrophil count of >0.5 × 10^9^/L for at least three consecutive days. The outcome classification, including GVHD, did not change over time in the current study. After collecting the data, acute and chronic GVHD were diagnosed and graded based on conventionally defined criteria [50,51]—namely, acute GVHD was defined as GVHD that developed within the first 100 days post-transplant, while the manifestations of GVHD occurring after day 100 were classified as chronic GVHD. The data using the updated criteria for the assessment of GVHD [52,53] were unavailable in the present cohort. The OS rate was defined as the number of days from transplantation to death from any cause. The DFS rate was defined as the number of days from transplantation to death from any cause or disease relapse or disease progression. Disease relapse was defined as the number of days from transplantation to disease relapse or disease progression. Transplant-related mortality (TRM) was defined as death without relapse. ALL patients who were alive at the last follow-up date were censored. The data on the causative microbes of infections, supportive care (including prophylaxis for infection and therapy for GVHD given on an institutional basis), and postmortem change in the cause of death were unavailable for this cohort.

The analysis was performed with Easy R (EZR) [54], which is a graphical user interface based on the R software program (The R Foundation for Statistical Computing, Vienna, Austria). The probability of OS was calculated using the Kaplan-Meier model and compared using the log-rank test. The probabilities of TRM, relapse, acute/chronic GVHD, and each cause of death were compared using the Gray test [55], and analyzed using a cumulative incidence model [56], considering relapse, death without relapse, death without acute GVHD, death without chronic GVHD, and death without each cause as respective competing risks.

OS, TRM, relapse, grades 2–4 acute GVHD, grades 3–4 acute GVHD, and chronic GVHD were calculated by using a multivariate Cox method and stepwise selection at a significance level of 5% to evaluate the HRs associated with the *HO-1* genotype. The variables included the recipient’s age at the time of transplantation, sex, pre-transplant CMV serostatus, disease characteristics (disease type, disease lineage, and disease risk at transplant), transplant characteristics (conventional or reduced-intensity conditioning) [57], donor characteristics (age, sex, sex compatibility, and ABO compatibility), cyclosporine versus tacrolimus, total nucleated cell counts harvested per recipient weight (TNC), and year of transplantation. We used the median value as the cutoff point regarding continuous variables. The independence of the variables used in the multivariate models was confirmed. The chi-squared test and Mann–Whitney test were used to compare between two groups. The Hardy–Weinberg equilibrium for the *HO-1* gene variation was tested using the Haploview program [58]. For both the univariate and multivariate analyses, two-tailed *p* values of <0.05 were considered to indicate statistical significance.

## 5. Conclusions

The findings of the present study suggested that the donor *HO-1* rs2071746 A/A or A/T genotype in the promoter region, which is expected to be highly inducible by HO-1, may improve survival outcomes in patients with high-risk hematologic malignancies who are undergoing unrelated BMT. *HO-1* rs2071746 genotyping in donors may therefore be a useful tool for evaluating pre-transplantation risk factors in patients with high-risk diseases that can form a basis for the appropriate tailoring transplantation strategies. Considering the plausible functional roles of this *HO-1* promoter SNP, it may be a candidate for future prophylactic and therapeutic strategies for complications after allo-HSCT for high-risk patients. Further studies are needed to confirm whether the findings of the present study can be extended to other stem cell sources or HLA-incompatible transplantation, as well as to the other ethnic groups.

## Figures and Tables

**Table 1:** Characteristics and heme oxygenase-1 (*HO-1*) genotypes of recipients and donors, according to the disease risk.

**Variable**	**All**	**High-Risk**	**Standard-Risk**	
	**Value**	**Value**	**Value**	***p***
Number of cases	593	232	361	
Recipient age, years, median (range)	33 (1–67)	34 (1–67)	34 (1–65)	
Donor age, years, median (range)	34 (20–57)	34 (21–50)	34 (20–57)	
Year of HSCT, median (range)	2000 (1993–2007)	2000 (1993–2007)	2000 (1993–2007)	
Recipient *HO-1* genotype, *n* (%)				0.03
A/A	125 (21)	40 (17)	85 (24)	
A/T	306 (52)	135 (58)	171 (47)	
T/T	162 (27)	57 (25)	105 (29)	
Donor *HO-1* genotype, *n* (%)				0.96
A/A	134 (23)	51 (22)	83 (23)	
A/T	308 (52)	122 (53)	186 (52)	
T/T	151 (25)	59 (25)	92 (25)	
Recipient sex, *n* (%)				0.55
Male	352 (59)	134 (58)	218 (60)	
Female	241 (41)	98 (42)	143 (40)	
Donor sex, *n* (%)				0.43
Male	374 (63)	142 (61)	232 (64)	
Female	218 (37)	90 (39)	128 (36)	
Recipient/Donor sex match, *n* (%)				0.54
Sex-matched	387 (65)	158 (68)	229 (64)	
Female/Male	114 (19)	41 (18)	73 (20)	
Male/Female	92 (16)	33 (14)	59 (16)	
Disease, *n* (%)				0.01
AML	197 (33)	112 (48)	85 (24)	
ALL	145 (24)	72 (31)	73 (20)	
MDS	82 (14)	0 (0)	82 (23)	
ML	64 (11)	29 (13)	35 (9)	
CML	101 (17)	16 (7)	85 (24)	
MM	4 (1)	3 (1)	1 (0)	
ABO matching, *n* (%)				0.97
ABO-matched	359 (61)	137 (59)	222 (62)	
Major mismatch	115 (19)	46 (20)	69 (19)	
Minor mismatch	95 (16)	40 (17)	55 (15)	
Bidirectional	16 (3)	6 (3)	10 (3)	
Missing	8 (1)	3 (1)	5 (1)	
Conditioning regimen, *n* (%)				0.86
Myeloablative	517 (87)	204 (88)	313 (87)	
Reduced intensity	69 (12)	26 (11)	43 (12)	
Missing	7 (1)	2 (1)	5 (1)	
Pretransplantation CMV serostatus, *n* (%)				0.23
CMV-positive recipient	373 (63)	142 (61)	231 (64)	
CMV-negative recipient	102 (17)	36 (16)	66 (18)	
Missing	118 (20)	54 (23)	64 (18)	
TNC, ×10^8^/kg, median (range)	7.7 (0.1–259)	7.7 (0.1–79.1)	7.7 (0.6–259)	

Abbreviations: AML, acute myeloid leukemia; ALL, acute lymphoblastic leukemia; MDS, myelodysplastic syndrome; ML, malignant lymphoma; CML, chronic myeloid leukemia; MM, multiple myeloma; HSCT, hematopoietic stem cell transplant; ABO, the “ABO” blood system; CMV, cytomegalovirus; TNC, total number of nucleated cells harvested.

**Table 2:** The results of the multivariate analysis of the association between the *HO-1* genotype and the clinical outcomes after transplantation (first part).

**Variable**		**OS**			**DFS**			**TRM**			**Relapse**	
	**HR**	**95% CI**	***p***	**HR**	**95% CI**	***p***	**HR**	**95% CI**	***p***	**HR**	**95% CI**	***p***
High-risk disease												
Donor *HO-1* genotype, A/T or A/A vs. T/T	0.72	0.50–1.04	0.08	0.64	0.44–0.93	0.02	0.66	0.36–1.19	0.16	0.83	0.51–1.35	0.44
Recipient *HO-1* genotype, A/T or A/A vs. T/T	0.91	0.62–1.32	0.61	0.99	0.68–1.45	0.98	1.03	0.57–1.85	0.92	0.99	0.60–1.64	0.98
Recipient age	0.99	0.97-0.99	0.004	0.99	0.98–1.00	0.02	0.98	0.96–0.99	0.006			
Standard-risk disease												
Donor *HO-1* genotype, A/T or A/A vs. T/T	1.05	0.71–1.54	0.80	0.91	0.56–1.47	0.70	1.04	0.59–1.82	0.89	0.71	0.40–1.22	0.20
Recipient *HO-1* genotype, A/T or A/A vs. T/T	1.39	0.93–2.08	0.10	1.30	0.79–2.17	0.28	1.15	0.66–2.00	0.62	1.41	0.76–2.63	0.28
Recipient age	0.97	0.96–0.98	<0.001	1.00	0.97–1.01	0.24	0.96	0.94–0.98	0.11	0.91	0.56–1.47	0.70
Donor age							0.97	0.94–1.01	0.11	1.33	0.80–2.22	0.27
CMV-positive recipient	1.22	0.77–1.92	0.39	0.58	0.36–0.94	0.03	0.47	0.26–0.82	0.008			
ABO major mismatch							1.04	0.54–200	0.90			
ABO minor mismatch	0.59	0.39–0.88	0.01				0.50	0.28–0.89	0.02			
ABO bidirectional							13513.5	5555.6–33333.3	<0.001			
Male donor/female recipient										1.00	0.58–1.75	0.99
Female donor/male recipient				2.27	1.05–5.00	0.04				2.22	1.03–4.76	0.04

Abbreviations: TRM, transplant-related mortality; HR, hazard ratio; CI, confidence interval; HSCT, hematopoietic stem cell transplant.

**Table 3:** The results of the multivariate analysis of the association between the *HO-1* genotype and the clinical outcomes after transplantation (second part).

**Variable**		**Grades 2–4 acute GVHD**			**Grades 3–4 acute GVHD**			**Chronic GVHD**	
	**HR**	**95% CI**	***p***	**HR**	**95% CI**	***p***	**HR**	**95% CI**	***p***
High-risk disease									
Donor *HO-1* genotype, A/T or A/A vs. T/T	1.10	0.61–1.96	0.76	1.03	0.41–2.78	0.88	0.91	0.51–1.59	0.72
Recipient *HO-1* genotype, A/T or A/A vs. T/T	1.09	0.62–1.92	0.76	1.00	0.40–2.50	1.00	0.63	0.37–1.08	0.09
Recipient age									
Donor age									
Conditioning regimen, MAC vs. RIC	3.03	0.94–10.0	0.06						
TNC	0.99	0.97–1.01	0.23						
Year of HSCT	0.59	0.35–0.98	0.04				0.97	0.94–1.00	0.10
Standard-risk disease									
Donor *HO-1* genotype, A/T or A/A vs. T/T	0.79	0.61–1.02	0.07	1.19	0.56–2.50	0.66	0.98	0.68–1.41	0.91
Recipient *HO-1* genotype, A/T or A/A vs. T/T	1.09	0.80–1.47	0.60	1.96	0.85–4.35	0.11	1.00	0.70–1.41	1.00
Recipient age							0.99	0.97–1.00	0.02
CMV-positive recipient	1.49	1.16–1.92	0.002						
Year of HSCT							1.61	1.14-2.27	0.008

Abbreviations: GVHD, graft-versus-host disease; MAC, myeloablative conditioning; RIC, reduced-intensity conditioning; TNC, total nucleated cell count harvested.

## Supplementary Materials

The following are available online at https://www.mdpi.com/2072-6694/12/2/424/s1. Figure S1: The Kaplan–Meier analysis of the overall survival rates (**A**) and the disease-free survival rates (**B**) after transplantation, according to the donor *HO-1* genotype in patients with standard-risk disease. Solid lines represent the donor A/A or A/T genotype. Dashed lines represent the donor T/T genotype. An asterisk denotes statistical significance (*p* < 0.05). In the standard-risk disease group, the donor genotype had no significant effect on the transplant outcomes, Table S1: The results of the univariate analysis of the association between the *HO-1* genotype and the clinical outcomes after transplantation (first part), Table S2: The results of the univariate analysis of the association between the *HO-1* genotype and the clinical outcomes after transplantation (second part).
